# Fecundity and human birth seasonality in Sweden: a register-based study

**DOI:** 10.1186/s12978-019-0754-1

**Published:** 2019-06-24

**Authors:** Johan Dahlberg, Gunnar Andersson

**Affiliations:** Department of Sociology, Stockholm University Demography Unit, Stockholm University, 106 91 Stockholm, Sweden

**Keywords:** Human birth seasonality, Register-based study, Fertility, Fecundability, Birth month, Sub-fertility, Second births, Time-to-pregnancy

## Abstract

**Background:**

It is well-established that couples’ fecundities vary widely. Each couple has a relatively constant monthly probability of conceiving, which can vary from zero to quite high. This underlying probability is usually expressed as the time (number of menstrual cycles) the couple requires to conceive. Couples with high fecundity will, on average, need fewer cycles than couples with low fecundity. It is also well-documented that almost all human populations exhibit seasonal variation in births. Most European countries show seasonal variation that usually peak in the spring and are the lowest during the last quarter of the year. The increasingly strong pattern of depressed birth rates in November and December is likely explained by the December–January cut-off threshold for Swedish pupils’ school entry and their parents increasing awareness of the negative effects on school outcomes for children who are juniors in the school-entry cohort they belong to. To actively plan births for a specific time of the year, couples need to have some knowledge of the time required for them to conceive.

**Methods:**

We use the duration between marriage of childless couples and first birth as a proxy measure of couples’ fecundity. Based on this time-to-pregnancy measure we study to what extent couples’ capacity to conceive affects the seasonality of their *second births*. We hypothesize that in a society with highly controlled fertility and a strong norm of having at least two children, sub-fertile couples will on average show less seasonal variation in second births. Sub-fertile couples, requiring more time to conceive the first time, will be less likely to try to target a desired birth month for their second child because doing so could jeopardize fulfilling their desired family size. We apply multinomial logistic regressions on 81,998 Swedish couples who married while being childless and subsequently gave birth to at least two children between 1990 and 2012, to investigate fecundity’s role in seasonal variation in second births.

**Results:**

We found that seasonal variation in second births was strongly associated with couples’ observed fecundity, measured as the duration between marriage formation and first birth. Our results support the hypothesis that sub-fertile couples, requiring more time to conceive the first time, show less seasonal variation in second births. The seasonal variations in second order births among couples with normal fecundity shows some similarities to traditional patterns as seen in agricultural and industrial societies of the past, with high numbers of births during the spring, and low numbers during the last quarter of the year. However, two important differences are notable. The characteristic Christmas peak in September has vanished, and the low birth rates in November and December come out much stronger than in the past.

**Conclusions:**

The birth seasonality among couples with normal fecundity are what we would expect if couples actively plan their births according to the cut-off date for Swedish pupils’ school entry. We argue that our findings support the notion that scheduled childbirth is a reality in contemporary sociality.

## Plain English summary

It is well-documented that almost all human populations exhibit seasonal variation in births. Increasingly strong pattern of depressed birth rates in November and December, in recent decades, is likely explained by the December–January cut-off threshold for Swedish pupils’ school entry and their parents increasing awareness of the negative effects on school outcomes for children who are juniors in the school-entry cohort they belong to. To actively plan births for a specific time of the year, couples need to have some knowledge of the time required for them to conceive. Couples’ fecundities vary widely. Each couple has a relatively constant monthly probability of conceiving, which can vary from zero to high. We use the duration between marriage of childless couples and first birth as a measure of couples’ fecundity. We hypothesize that in a society with highly controlled fertility and a strong norm of having at least two children, sub-fertile couples will on average show less seasonal variation in second births. Sub-fertile couples, requiring more time to conceive the first time, will be less likely to try to target a desired birth month for their second child because doing so could jeopardize fulfilling their desired family size. Studying 81,998 Swedish couples who married while being childless and subsequently gave birth to at least two children between 1990 and 2012, we found that seasonal variation in second births was strongly associated with couples’ observed fecundity. Seasonal variation in the physiological ability to reproduce and cultural behaviours linked to the likelihood of sexual intercourse may still matter for when under the year births occur. However, our study supports the notion that the impact of these factors is relatively weak in contemporary societies. We argue that our findings support the notion that scheduled childbirth is a reality in contemporary sociality.

## Background

Researchers have repeatedly reported strong seasonal variation in human births and that the patterns vary across geographic regions [1]. Human birth seasonality has been linked to such exogenous factors as photoperiod, climate, nutrition, cultural, and sociodemographic factors. Seasonal variations in temperature, daylight, and female nutritional status have been linked to seasonal variations in birth via semen quality, ovulation frequency, and menstrual cycle length [2, 3]. The mother’s social-demographic characteristic, such as maternal education, age, and parity, have all been shown to affect the time of year in which births occur [4]. Factors associated with the probability of intercourse, such as major secular and religious holidays, have also been shown to cause seasonality in human births [5]. However, it has been argued that in a society with a below-replacement birth rate and highly controlled fertility due to efficient contraception, active choices and behaviours associated with individual sociodemographic characteristics override the role of factors that influence the physiological ability to reproduce and cultural behaviours that affect the likelihood of sexual intercourse. In a society with more active planning of the timing of childbirth, optimal birth months for parents’ career building and the child’s school outcome may affect seasonal variations more than cultural factors associated with the probability of intercourse. [4, 6–8]. In Europe, pregnancy planning is not evenly distributed over the year, with the summer season typically being the preferred time for starting pregnancy [9].

To actively plan births for a specific time of the year, couples need to have some knowledge of the time required for them to conceive. Previous research has shown that mothers who re-partnered between births displayed less seasonal variation than mothers who resumed childbearing with the same partner [4]. A possible conclusion of these findings is that mothers with no re-partnering interruptions in their childbearing careers are better able to plan the timing of their next birth because they have better knowledge about their level of fecundity.

It is well-established that couples’ fecundities vary widely. Time-to-pregnancy (TTP) is considered one of the most direct methods to measure natural fecundity in humans [10]. Each couple has a relatively constant monthly probability of conceiving, which can vary from zero to quite high. This underlying probability is usually expressed as the time (number of menstrual cycles) the couple requires to conceive. Couples with high fecundity will, on average, need fewer cycles than couples with low fecundity [11, 12]. Thus, if scheduled childbirth is a reality, the most fecund couples will more often conceive within their preferred time window, but those who are sub-fertile may not succeed [9]. Swedish couples’ preferred time for starting pregnancy has not been studied. However, in several Western European countries the spring is often the most preferred time for births [9, 13].

In the current study, we use the duration between marriage of childless couples and their first birth as a proxy measure of couples’ fecundity. Based on this TTP measure we study to what extent couples’ capacity to conceive affects the seasonality of their *second births* as reflected in the birth month of their second children. No previous study has addressed birth seasonality in relation to couples’ fecundity using a TTP measure. We hypothesize that in a society with highly controlled fertility and a strong norm of having at least two children, sub-fertile couples will on average show less seasonal variation in second births. Sub-fertile couples, requiring more time to conceive the first time, might be less likely to try to target a desired birth month for their second child because doing so could jeopardize fulfilling their desired family size. Evidently, they are also less able to achieve any specific target they may try to reach.

We apply multinomial logistic regressions on 81,998 Swedish couples who married while being childless and subsequently gave birth to at least two children between 1990 and 2012, to investigate fecundity’s role in seasonal variation in second births.

Contemporary Sweden is a particularly good setting to study this issue. With our study design, the studied population needs to meet at least three criteria. First, it is vital that most couples who marry while still being childless also start trying to conceive in conjunction with their marriage. In Sweden, few couples enter marriage without previously living together [14], marriage is mainly considered an act of showing that the partners are committed to their existing relationship. For most couples in Sweden who have no premarital children, marriage is not the starting point of a relationship but the starting point of the next stage in family formation, namely parenthood [15]. Second, it is necessary for our study design that the duration between couples’ first birth and when they start trying to conceive a second time is not affected by other intervening factors such as the couple’s economic situation or parents’ career building. In the Swedish case, these factors are arguably less intrusive than in other contexts. Sweden has one of the world’s most generous systems of job security and economic benefits for parents [16]. Sweden also has a strong norm of families having at least two children [17], and Swedish women show on average relative short intervals between their first and second births [18]. Third, the seasonal variations in the studied population should not be caused by variations in female nutritional status or cultural factors, such as the impact of major secular or religious holidays, that affect the probability of intercourse. Contemporary Sweden does no longer show any seasonal variability in births that can be attributed to its secular or religious holidays. The characteristic Christmas effect that used to be visible in the high numbers of births in September vanished in the 1980s [4]. Since the 1990s, the most pronounced pattern of seasonality in Swedish births is a steep decline in births during the last quarter of the year. This pattern is observed among mothers of all educational levels but is significantly more pronounced among highly educated mothers [4]. This strong pattern of depressed birth rates in November and December is likely explained by the December–January cut-off for Swedish pupils’ school entry and their parents’ increasing awareness of the negative effects on school outcomes for children who are juniors in their school-entry cohorts [19]. Well-educated parents tend to be more likely to invest in their children’s education [20] and thus be more concerned about such matters. These factors are strong reasons to believe that active planning of the timing of childbearing causes the observed seasonality of births in contemporary Sweden.

## Material and methods

### Study population

Data were retrieved from the Swedish multigenerational register, with information on all Swedes born from 1932 onwards who have been registered as residents in Sweden at any time since 1961. The data contain highly accurate information on vital events such as childbirths and changes in marital status. The information used in the present study includes all Swedish couples who married and gave birth to at least two children anytime between 1990 and 2012. Women and men who had previously been married, had children from a prior relationship, or re-partnered between births one and two were excluded. Women older than age 40 at marriage were also excluded due to the risk that their relatively high age would affect the duration between marriage and first birth. Couples who had ever adopted, had multiple births at first pregnancy, or separated before a second birth were also excluded from the analysis. The study only analysed Swedish-born men and women as the meaning of marriage and its relation to childbearing decisions might differ among individuals from different cultural backgrounds. A small number of parents who experienced their firstborn child’s death were also excluded from the analysis. We included couples who had their first child up to five years after their marriage. Finally, couples who gave birth less than nine months following the marriage were excluded as we only want to study couples who commence their childbearing careers subsequent to marriage formation. A total of 81,998 Swedish couples met all these criteria. As we only include couples with no prior marriage or children and assume that these men and women have not previously actively been trying to conceive, we analyse couples that, to a large extent, had no prior knowledge about their levels of fecundity.

### Sub-fertility and birth month

The dependent variable in this study is the second child’s month of birth. We report the differences between the expected and observed probability of second childbirth for each month in the calendar year for couples with different levels of observed fecundity.

TTP, the duration from the time a couple starts attempting to become pregnant until they succeed, is the most widely used measure of natural fecundity in humans. Sub-fertility is usually defined as a failure to conceive after one year (or sometimes two years) of trying [21]. In the present study, we use time between marriage and first birth as a TTP measure of couples’ fecundity. Because we only include newly married childless men and women, the time it takes the couple to give birth should on average capture the couples’ underlying fecundity. In our study, couples who gave birth to a first child between nine months and two years after their marriage was considered to have normal fecundity. Couples who had a longer duration than two years between marriage formation and first childbirth are considered sub-fertile.

### Maternal education, age and month of marriage

In addition to the main independent variable that measures fecundity, we also included control variables for maternal education and mother’s age at first birth. Fertility is to a considerable extent negatively correlated with female age [22], making maternal age a potential confounding factor in our analysis. Older women may be less likely to target a specific birth month due to the risk of not realizing their desired family size. However, in Sweden, it has been found that highly educated women who postpone motherhood also are more likely to have shorter intervals between first and second births [23]. Previous research has also shown that highly educated women, who are on average older when entering motherhood [24], are also more likely to avoid childbirth at the end of the year [4].

### Statistical analysis

Multinomial logistic regressions were used to calculate predicted probabilities of giving second birth in each calendar month. The results are reported as the observed versus expected probability of childbirth for each month. The baseline (expected probability) is expressed as 100 births per month. Each month’s number of days was taken into consideration (February is assumed to have 28.25 days on average). Using multinomial logistic regression rather than simply comparing expected and observed numbers of births allows for multivariate analyses with the controls for potentially confounding factors. Two-tailed *P*-values < 0.05 were considered markers of statistical significance. All statistical analyses were performed utilizing STATA 15 (StataCorp. 2017. Stata Statistical Software: Release 15. College Station, TX, USA: StataCorp LLC).

## Results

**Fig. 1:**
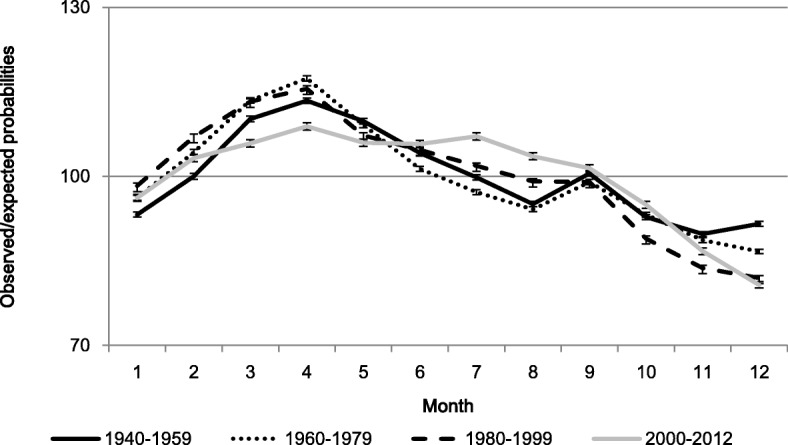
Seasonal variation in births in Sweden by year. (error bars are 95% CI). *N* = 6,768,810. Reprinted from Dahlberg and Andersson. Changing Seasonal Variation in Births by Sociodemographic Factors: A Population-Based Register Study. Human Reproduction Open. 2018 [4] by permission of Oxford University Press/Human Reproduction Open

Current patterns in Swedish birth seasonality are a relative new phenomenon. Figure 1 shows the seasonal variation in live births in Sweden between 1940 and 2012. At the end of the twentieth century, Swedish birth rates showed the typical seasonal variation with high numbers of births during the spring and low numbers of births during the last quarter of the year. However, during the new century, the seasonal variation was reduced to a situation where there was only minor variation in birth rates between February to September. The pattern of low birth rates at the end of the year remained and even gained in magnitude. Additionally, the previously characteristic Christmas peak in September births has vanished over the last three decades. The finding that holiday effects in birth seasonality have vanished or weakened is consistent with an assumption that cultural behaviours associated with the likelihood of intercourse play a much less significant role in contemporary childbearing behaviour in Sweden. Concurrent, clear declines in birth seasonality have occurred in several other Western European countries [25–31].

**Fig. 2:**
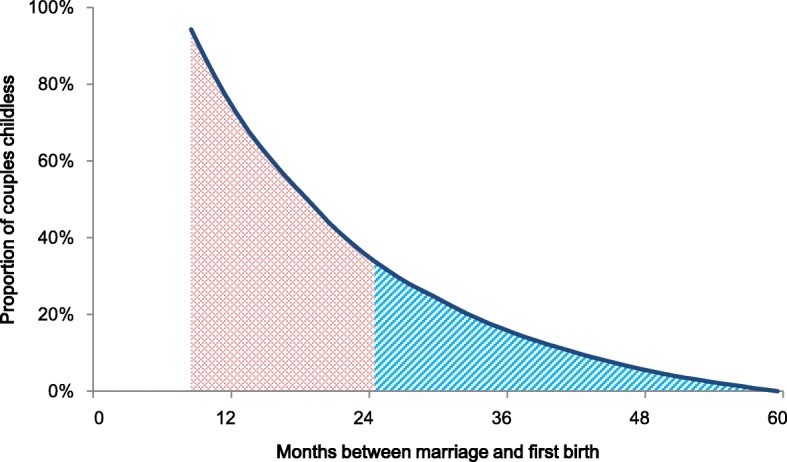
Distribution of couples’ duration between marriage and first birth

The distribution of Swedish couples’ achieved duration time between marriage and first birth is shown in Fig. 2. The pink area under the blue line corresponds to the proportion of couples with normal fecundity (first birth between nine months and two years after marriage). The blue area below the line corresponds to the proportion of couples that are sub-fecund. Approximately two-thirds of couples had a first birth between nine and 23 months after their marriage.

**Fig. 3:**
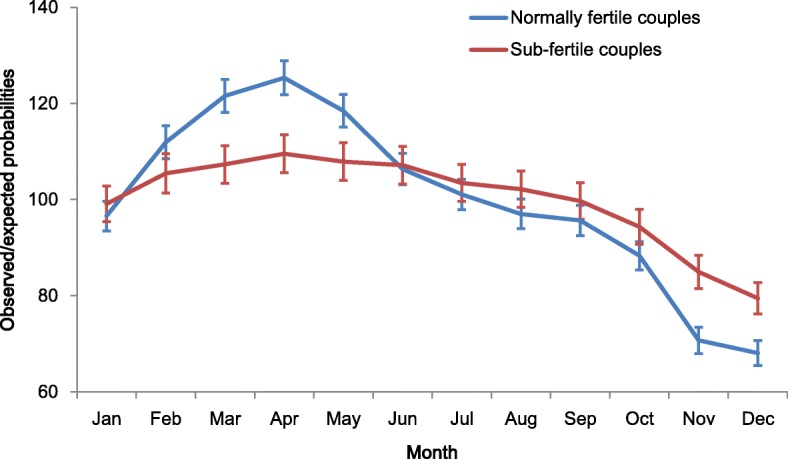
Seasonal variation in second births by couples’ fecundity. (error bars are 95% CI). *n* = 81,998

The differences between the expected and observed probabilities of second births for each month by couples’ fecundity are shown in Fig. 3. The solid blue line represents the observed number of births for each month for couples with normal fecundity, and the red line represents the observed number of births for sub-fecund couples.

A clear difference emerges in seasonality of live births between more and less fertile couples. Normally fertile couples show a typical seasonal variation of Sweden with high numbers of births during the spring and low numbers during the last quarter of the year. Among couples with normal fecundity, we observe around 120 births in March, April and May per 100 expected births. Sub-fertile couples, on the other hand, show significantly less seasonality in second births. Between January and September, sub-fertile couples show only minor seasonal variation in births. Normally fertile and sub-fertile couples show depressed birth rates in November and December, but the depressed second birth rates are significantly amplified among the more fertile couples. Couples with normal fecundity show around 70 births in November and December per 100 expected births. The same figure for sub-fertile couples is around 80.

All meaningful differences reported are statistically significant. We also carried out two sensitivity analyses. First, we tested if the results changed significantly if we increased the studied population by included couples who gave birth to their first child up to seven years after marriage. However, this did not change the results in any way. Second, we carried out separate analyses for different years of marriage formation to see if the difference in seasonal variations in second births between normal and sub-fertile couples had changed during the period studied. However, we did not detect any meaningful changes in patterns.

## Conclusions

We found that seasonal variation in second births was strongly associated with couples’ observed fecundity, measured as the duration between marriage formation and first birth. The results support the hypothesis that sub-fertile couples, requiring more time to conceive the first time, show less seasonal variation in second births. Normally fertile and sub-fertile couples all show decreased birth rates at the end of the year, but the pattern was significantly stronger among the more fertile couples. Normally fertile couples also displayed a substantial peak in births in the spring, but sub-fertile couples displayed almost no variations in second births over the first nine months of the year. The results are in line with previous research that has shown that mothers who re-partnered between subsequent births displayed less seasonal variation than mothers who resumed childbearing with the same partner [4]. Mothers who re-partnered between subsequent births should on average have less knowledge of her and her new partner’s likelihood to conceive and thus have a lower ability to target a desired birth month.

TTP is not a measure of fecundability for each individual couple, but the best measure of couple fecundity that we have at the population level. All types of TTP data have their advantages and disadvantages. Sampling, memory, and truncation biases are the main disadvantage with traditional TTP measures [32]. A prospective study design is often preferable, but it is difficult to obtain representative sample of couples that try to conceive [33]. In retrospective studies, couples are asked to recall their TTP with risks of unreliable time-specific information about behaviour and risk factors [10]. New methods of measuring couple fecundability have been called for [32]. By utilizing Swedish register data with high-quality information on couples’ characteristics and their childbearing behaviour, we used the duration between marriage of childless couples and their first birth as a TTP measure. This provided large-scale data with non-biased information on couples’ childbearing outcomes. The drawback with our measure is that we have no information on issues such as the timing of contraceptive use or the frequency of intercourse.

In our data, two-thirds of couples had a first birth between nine and 23 months since marriage formation. The median TTP was 19 months. In general, survey-based TTP estimates - both retrospective and prospective - tend to report higher proportions of couples with normal fecundity and lower proportions with sub-fecundity. However, the median TTP in our study corresponds quite well with other TTP measures [34–36].

For a correct interpretation of our results, we note that the proxy we defined for couples’ fecundity builds on two assumptions. First, that couples start trying to conceive in conjunction with their marriage, and second, that couples have no prior knowledge about their fecundity when getting married. As already mentioned, marriage in Sweden is mainly considered an act of commitment in an existing relationship, and childless couples who get married mainly enter the next stage in their family formation, namely parenthood. Some of the men and women in the studied population may already have tried to become a parent but did not succeed and thereby had some knowledge about their level of fecundity. However, by excluding men and women who had previously been married or had children from a prior relationship, we minimize this risk. Restricting the study population as we did, our results should be robust enough to support the hypothesis that sub-fertile couples are less likely to target a specific time of the year for their second child.

These findings support the assumption that couples to quite some extent plan their childbearing according to specific desired birth months. Our results do not only show stronger seasonality in second births among couples with normal fecundity than for sub-fertile couples. They also show that the birth seasonality among couples with normal fecundity are what we would expect if couples actively plan their births according to the cut-off date for Swedish pupils’ school entry.

To some extent, the seasonal variations in second order births among couples with normal fecundity shows similarities to traditional patterns as seen in agricultural and industrial societies of the past, with high numbers of births during the spring, and low numbers during the last quarter of the year. However, two important differences stand out: The characteristic Christmas peak in September has vanished, and the low birth rates in November and December come out much stronger than in the past.

Sweden is often considered one of the most gender equal countries in the world where mothers’ labor force participation, shared parental leave, and dual-earner dual-carer couples are the norm [37, 38]. In such a context, patterns in parental leave use may also matter for Swedish parents’ planning of their childbirths. For many couples a spring birth may be perceived as desirable as it is followed by a pleasant summer period in parental leave with the new-born, which in turn can be shifted from the mother to the father during the second summer of the child’s life: Swedes are entitled to about one and half year of paid parental leave, of which the father on average take about a quarter [39].

We cannot yet rule out, that seasonal variation in nutrition, daylight and cultural behaviours linked to the likelihood of sexual intercourse still matter for when under the year births occur. However, our study supports the notion that the impact of these factors is relatively weak in contemporary societies, and that increased individual autonomy has contributed to more conscious planning of the timing of childbirths. In contemporary societies with below-replacement and highly controlled fertility, active choices matter more for childbearing than the physiological ability to reproduce. Our study thus supports the notion of scheduled childbirths [13]. Active choices and behaviours associated with individual sociodemographic characteristics seem to have overridden the importance of factors that mattered for the seasonality of childbearing in the past.

## Data Availability

The article is based on individual level demographic data, derived from contemporary Swedish registers (containing detailed socioeconomic information on the complete current population of Sweden). This has been approved by a Swedish national ethical review board and data have been made available by Statistics Sweden. Due to the sensitive nature of these data, they cannot be shared without an application to the Swedish ethical review board. As such, the underlying micro-level data cannot be made directly available in a public depository. However, we are helpful in providing access to our data for any researcher with the ethical permission to use the underlying data.

## Abbreviation

TTP: Time-to-pregnancy
